# Supplementary figures and images for: A cell wall synthase accelerates plasma membrane partitioning in mycobacteria
Source: eLife. 2023 Sep 4;12:e81924. doi: 10.7554/eLife.81924 (PMC10547480; doi:10.7554/eLife.81924)

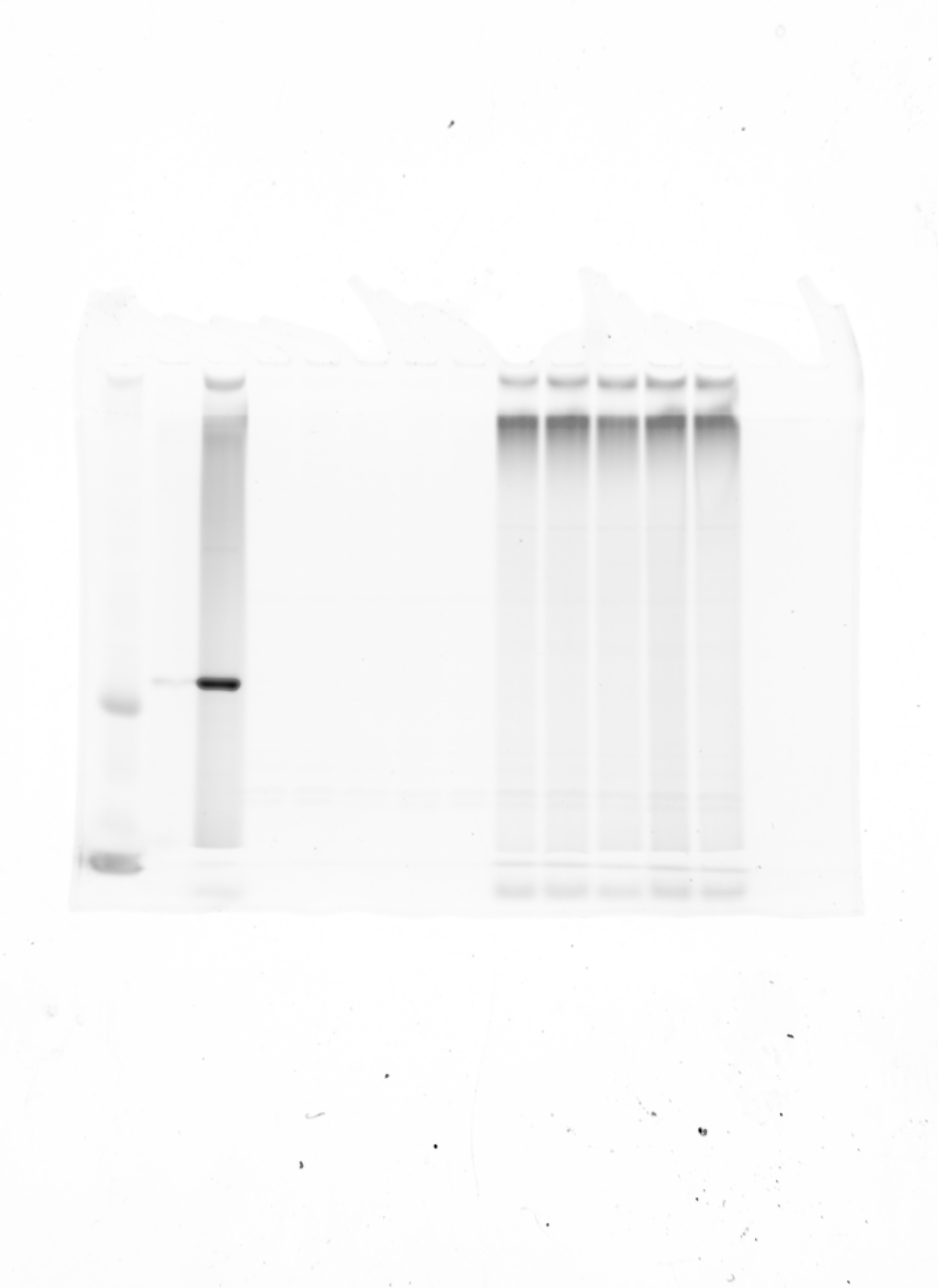

Supplement: Figure 6—figure supplement 2—source data 1. [file elife-81924-fig6-figsupp2-data1.zip › Siegrist_15-07-2022-RA-eLife-81924R2_Figure_6_figure_supplement_2_Source_data.tif]

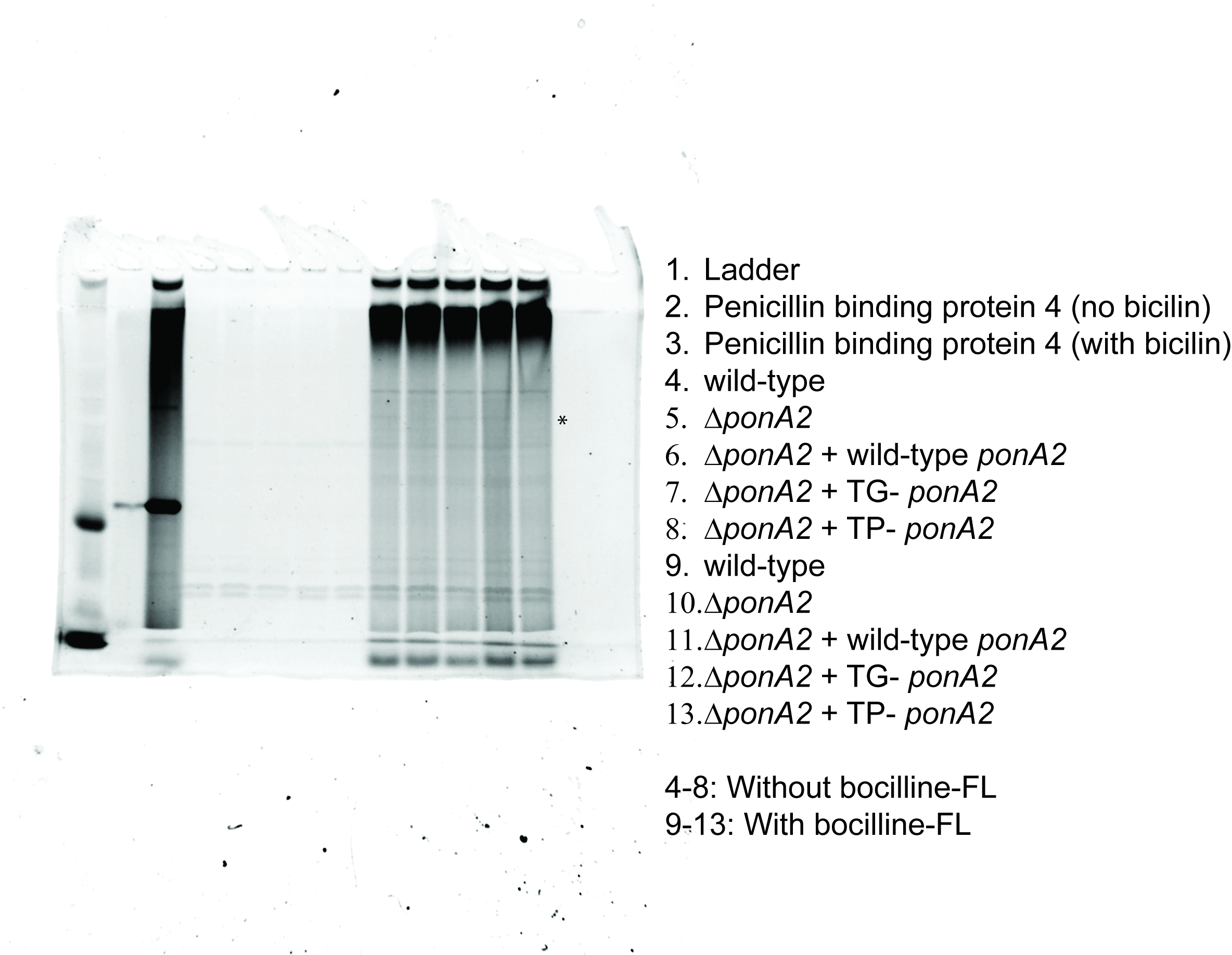

Supplement: Figure 6—figure supplement 2—source data 1. [file elife-81924-fig6-figsupp2-data1.zip › Uncropped_blot_of_Figure_6-figure_supplement_2.tif]
