# Supplementary material for: A cell wall synthase accelerates plasma membrane partitioning in mycobacteria
Source: eLife. 2023 Sep 4;12:e81924. doi: 10.7554/eLife.81924 (PMC10547480; doi:10.7554/eLife.81924)
Supplement: Supplementary file 1. [file elife-81924-supp1.docx]

Supplementary Table. Primers used in this study.

| Name | Description | Sequence |
| --- | --- | --- |
| A980 | ponA2_Fw | AACGCGTGCGGCCGCGGTACTTTGGGACGGCCGTGCAGTC |
| A981 | ponA2_Rev | GCAGCTGGATCCATGGATATGTCACGGATCGATCCTTGGC |
| A995 | ponA2_E193T(TG-)_Fw | CATCaccGACAAGCGGTTC |
| A996 | ponA2_E193T(TG-)_Rev | GAgACGATCGCGAGCTTC |
| A997 | ponA2_S473A(TP-)_Fw | GGCgccGTGTTCAAGATC |
| A998 | ponA2_S473A(TP-)_Rev | CGCACCGTTGCCCACCAG |

Lowercase letters indicate where mutations were introduced.
